# Radiosensitization of breast cancer cells using AS1411 aptamer-conjugated gold nanoparticles

**DOI:** 10.1186/s13014-021-01751-3

**Published:** 2021-02-10

**Authors:** Somayeh Sadat Mehrnia, Bijan Hashemi, Seyed Javad Mowla, Maryam Nikkhah, Azim Arbabi

**Affiliations:** 1 Department of Medical Physics, Faculty of Medical Sciences, Tarbiat Modares University, P.O. Box: 14115-331, Tehran, Iran; 2 Department of Molecular Genetics, Faculty of Biological Sciences, Tarbiat Modares University, Tehran, Iran; 3 Department of Nanobiotechnology, Faculty of Biological Sciences, Tarbiat Modares University, Tehran, Iran; 4 Department of Radiotherapy, Imam Hossein (A.S.) Hospital, Shahid Beheshti University of Medical Science, Tehran, Iran

**Keywords:** Gold nanoparticles, Radiosensitizer, Aptamer AS1411, Breast cancer cells, Mammosphere, 4 MeV electron beam, Radiation therapy

## Abstract

**Background:**

Gold nanoparticles (GNPs) have been used to sensitize cancer cells and enhance the absorbed dose delivered to such cells. Active targeting can provide specific effect and higher uptake of the GNPs in the tumor cells, while having small effect on healthy cells. The aim of this study was to assess the possible radiosensitiazation effect of GNPs conjugated with AS1411 aptamer (AS1411/GNPs) on cancer cells treated with 4 MeV electron beams.

**Materials and methods:**

Cytotoxicity studies of the GNPs and AS1411/GNPs were carried out with MTT and MTS assay in different cancer cell lines of MCF-7, MDA-MB-231 and mammospheres of MCF-7 cells. Atomic absorption spectroscopy confirmed the cellular uptake of the gold particles. Radiosensitizing effect of the GNPs and AS1411/GNPs on the cancer cells was assessed by clonogenic assay.

**Result:**

AS1411 aptamer increased the Au uptake in MCF-7 and MDA-MB-231 cells. Clonogenic survival data revealed that AS1411/GNPs at 12.5 mg/L could result in radiosensitization of the breast cancer cells and lead to a sensitizer enhancement ratio of 1.35 and 1.66 and 1.91 for MCf-7, MDA-MB-231 and mammosphere cells.

**Conclusion:**

Gold nanoparticles delivery to the cancer cells was enhanced by AS1411 aptamer and led to enhanced radiation induced cancer cells death. The combination of our clonogenic assay and Au cell uptake results suggested that AS1411 aptamer has enhanced the radiation-induced cell death by increasing Au uptake. This enhanced sensitization contributed to cancer stem cell-like cells to 4 MeV electron beams. This is particularly important for future preclinical testing to open a new insight for the treatment of cancers.

## Background

Many efforts in radiation oncology have focused on approaches that aim to preferentially sensitize tumors to radiation and minimizing radiation effects on normal tissues [1, 2]. Multimodal approach for cancer therapy is wildly used to improve therapeutic index and enhance tumor response to ionizing radiation by using radiosensitizer agents like high atomic number elements [3–6]. Development of nanotechnology techniques provides the possibility of using nanomaterials in medicine especially in the detection and treatment of cancer. Nanomaterials such as gold nanoparticles (GNPs) have high atomic number (Z = 79) and preferential mass energy absorption compared to soft tissues; thus present radiosensitizing properties and potentially improve tumor control, reduce the side effects and increase the patients survival when compared to radiotherapy alone [5, 7–9]. Additionally, GNPs are relatively easy to synthesize in a range of sizes, and have been shown to passively accumulate in tumors through their enhanced permeability and retention (EPR) effect [10]. A number of biologically active molecules such as proteins, DNA or oligonucleotides can be bound to GNPs that make it capable of targeting, detection and cure of cancer cells. Coating GNPs with DNA, RNA or oligonucleotides improves its stability and persistence in circulation and allowing its greater accumulation in the tumor tissues. Most targeting ligands are specific to over-expressed cancer cells membrane receptors which provide specific binding and sometimes the advantage of receptor-mediated internalization into tumor cells [11–13].

AS1411 is a 26-base guanine-rich oligonucleotide, commonly known as anti-nucleolin aptamer, which forms a stable dimeric G-quadruplex structure to specifically bind the target nucleolin receptors over-expressed on cancer cells [14, 15]. Since there is a relative lack or lower levels of the nucleolin receptors on the plasma membrane of normal cells, nucleolin could be regarded as a tumor biomarker to distinguish cancer cells from normal ones [14]. Thus, the AS1411-nucleolin specific interaction could be utilized as a strategy to mediate highly specific and effective targeting of therapeutic agents to cancer cells [16, 17]. It has been demonstrated that the AS1411aptamer could enhance the uptake of certain nanoparticles in cancer cells such as MCF-7 and Hela cell [16].

As a new concept, several evidence suggest that there is a subpopulation of tumor cells called cancer stem/progenitor cells that are more resistant to radiation and responsible for cancer recurrence after treatment [18–21]. Accordingly, a new insight in cancer treatment is the removal of cancer stem cells to prevent its’ recurrence and improve the treatment efficacy. Cancer stem/progenitor cells can be enriched by proliferating cells in serum-free, growth factor-enriched conditions as 3-dimentional mammosphere culture [22, 23].

Briefly, several studies have showed that GNPs can act as radiosensitizer. Increasing the uptake of GNPs by cancer cells results in the increased efficiency of radiotherapy. Combining of this approach with enhanced GNPs delivery to cancer cells by AS1411 aptamer will duplicate the effectiveness of radiotherapy. This report bolds the effect of combination of the GNPs mediated radiosensitizing with AS1411 aptamer mediated cancer cell targeting in radiotherapy by 4 MeV electron beams in breast cancer cell cultures. We also showed the efficiency of AS1411/GNPs in radiosensitizing of breast cancer cells grown as mammospheres as a model for cancer stem- like cells (CSC) enriched culture.

## Methods

### Preparation of GNPs and AS1411 conjugated GNPs

GNPs were synthesized based on a previous report [24] with some modifications. Briefly, all experimental glasswares were thoroughly washed in Aqua Regia (3 parts HCl and 1 part HNO3), and all solutions were prepared using 18-MΩ-deionized water. Fifty milliliter of HAuCl_4_ (0.25 mM; Sigma- Aldrich) was reduced with sodium citrate (1% w/v, 2 mL) by boiling and vigorous stirring for 10 min. The resultant reddish-purple suspension was cooled, sterile-filtered and stored in glass bottles at 4 °C. The quality of GNPs was checked using UV–Visible (LAXCO, alpha 1900s, US), Spectrophotometer dynamic light scattering (DLS, Malvern Zetasizer Nano ZS, UK) and transmission electron microscopy (TEM: 80 kV, EM10C, Zeiss, Germany).

Thiolated AS1411 Aptamers (5′GGTGGTGGTGGTTGTGGTGGTGGTGGTTTSH-3′) (BIORON GmbH, Germany) were dissolved in 18-MΩ deionized water. All mixing processes were performed under the laminar flow hood to prevent any contamination. Conjugation of oligonucleotides to the GNPs was achieved using a method based on a protocol reported by Mirkin et al. [25]. Briefly, AS1411 aptamers [4 nmol] were reduced by 1 h incubating with tris-(2-carboxyethyl)-phosphine hydrochloride (TCEP: 10 mM; Invitrogen) at room temperature followed by precipitation with ethanol. Then they were added to 10 nm colloidal GNPs (50 mg/L, 3 mL) with shaking and incubating at room temperature for 24 h. The particles were slowly supplemented at room temperature by adding 10X phosphate buffered saline every 12 h until 1X concentration was reached in a period of 48 h. Following incubation, the GNPs complexes were divided into ten 1.5 mL tubes and centrifuged at 12,000 g for 45 min to separate the conjugated GNPs and unconjugated oligonucleotides. To sterilize, the conjugated GNPs constructs were filtered by 0.2 µm PTFE filter before centrifugation. The hydrodynamic size and the quality of AS1411/GNPs were evaluated using UV–Visible spectroscopy and dynamic light scattering (DLS). Prior to use, all nanoparticles were washed by repeated cycles of centrifugation to acquire the excess reactants elimination. The absence of any aggregation resulted from washing steps was checked by UV–visible spectra before and after centrifugation; while the band shape and position remained unchanged for all the cases.

To measure the coupling efficacy, the concentration of unconjugated oligonucleotides in supernatant was measured. Molar ratio of aptamer to GNP was calculated based on the changes in molar concentration of aptamer in supernatant and the molar concentration of GNP.

### Cell culture

Breast cancer cell lines of MCF-7 (IBRC C10682, Iranian Biological Resource Center) and MDA-MB-231 (IBRC C10684, Iranian Biological Resource Center), and human normal fibroblast cell line of HFSF-PI3 (C167, Pasteur Institute of Iran) were cultured in Dulbecco’s modified Eagle’s medium (DMEM, Gibco) supplemented with penicillin/streptomycin (1%; Invitrogen), and fetal bovine serum (FBS: 10%, Gibco) at 37 °C in an atmosphere of 5% CO_2_ incubator.

The study of cancer stem cells has been made easier through an in vitro enrichment technique called mammosphere culture [22]. MCF-7 Cells at a density of 1 × 10^3^ cells/ml were placed in serum-free DMEM supplemented with Epidermal Growth Factor (EGF: 20 ng/ml; RoyanBiotech), basic Fibroblast Growth Factor (bFGF:20 ng/ml; RoyanBiotech), Non-Essential Amino Acids (NEAA: 0.1 mM; Gibco), B27 (1X; Invitrogen), penicillin/streptomycin (1%; Invitrogen), in ultralow attachment plates (Costar, USA). Approximately after 5 days the spheres were collected by gentle centrifugation (2 min at 500 g), dissociated with trypsin/EDTA and mechanically disrupted and after centrifugation (5 min, 2500 g) used for consequent experiments.

### Real time PCR

The expression of OCT4-a as the main transcriptional factor that exerts key roles in the maintenance of self-renewal and pluripotency in human embryonic stem cells [26–30] was studied by real time PCR. Total RNA was isolated from adherent or mammospheres cultures using TRIzol reagent (Invitrogen Life Technologies, USA) according to the manufacture instructions. As a positive control, the RNA of human EC cell line NTERA2cl (NT2) was used. After the treatment with DNaseI (Thermo Fisher Scientific, Waltham, MA: USA), cDNA synthesis was performed by the RevertAid™ Reverse Transcriptase kit (Fermentas, GMBH, Germany) and oligo-dT primer (GeneOn, Germany) as instructed by the companies. qPCR was done with specific primers for Oct4-a (forward: 5′-CTTCTCGCCCCCTCCAGGT-3′, reverse: 5′-AAATAGAACCCCCAGGGTGAGC-3′) and β2M (forward:5^′−^ GGGTTTCATCCGACATTG-3′ reverse: 5′-TGGTTCACGGCAGGCATAC-3′) as internal control. The amplification was performed using qPCR master mix (SYBR-Green: Ampliqon, Herlev, Denmark) with a qPCR instrument (Step One, Applied Biosystems, Korea). The data were evaluated as 2^−ΔΔCt^ values (Ct indicates the cycle of threshold). All Ct values calculated from the target genes were normalized to B2M as the reference gene, and the fold change to the control was calculated for the comparison. The resultant quantitative PCR products were resolved on 1% agarose gel and stained with ethidium bromide.

### Evaluation of cell toxicity

To determine the cell toxicity following treatment with various concentrations of GNPs and/or AS1411/GNPs conjugate, the cells viability in adherent cells were assessed using the MTT (3-(4,5-Dimethylthiazol-2-yl)-2,5-diphenyltetrazolium bromide, Sigma-Aldrich) assay. Cells were seeded in 96-well plates and 24 h later were treated with different concentrations of the GNPs (0, 12.5, 25 and 50 mg/L) for an additional 24 h. The cytotoxicity of the AS1411/GNP conjugates and an equivalent amount of the GNPs alone, was assessed by adding the MTT solution in a fresh medium for 4 h. The treated cells were collected in DMSO (Dimethyl sulfoxide**)** and placed on a shaker for 5 min. The absorbance of final solution was measured at 540 nm using a 96-well plate reader (ELISA-Reader, Hyperion, Canada). The results were normalized to the control and presented as the percentages of absorbance for untreated control cells. Three independent experiments were done for each data point.

For evaluating the MCF-7 mammosphere cells viability, the MTS (3-(4,5-dimethylthiazol-2-yl)-5-(3-carboxymethoxyphenyl)-2-(4-sulfophenyl)-2H-tetrazolium) assay was used. Seven days after cell seeding in low attachment 96-well plates (Costar) in a density of 1000 cell/mL, the grown mammospheres were exposed to 12.5 mg/L of the GNPs or AS1411/GNPs. After 24 h, the cytotoxicity of the GNPs or AS1411/GNPs was assessed by adding the MTS solution in a fresh medium for 4 h. The absorbance of final solution was measured at 490 nm using a 96-well plate reader (ELISA-Reader, Hyperion, Canada). The results were normalized to the control. Three independent experiments were done for each data point.

### GNPs uptake assay

The uptake of GNPs and AS1411/GNP by cancer cells was quantified by atomic absorption spectroscopy (AAS) (SHIMADZU AA-670G, Japan). For AAS measurements, 70,000–100,000 cells were seeded per well in 24-well plates in 0.5 mL of a complete culture medium; 24 h post seeding, (when the plated cells became 70–80% confluence) the cells were treated for another 24 h with 6, 12.5, 25 and 50 mg/L of the GNPs or AS1411/GNPs solutions. At the end of the exposure time, the medium was removed, the wells were washed for 3 times with phosphate-buffered saline (PBS), and exposed to 0.05% Trypsin–EDTA for 2–3 min. A fresh complete medium was added and the cells were collected for counting. Then, each sample was collected in a separate tube and the amount of Au was analyzed by AAS after mineralization with Aqua Regia and sonication. Three independent experiments were carried out and the results were calculated as Au concentration (ng/cell).

To show AS1411 aptamer uptake in the cancerous MCF-7 cells and normal cells (Fibroblast cells), we utilized the AS1411 aptamer with 5′-6FAM modification. The cells were seeded in 4-well plates. After 24 h treatment with 1 µM AS1411 aptamer, the cells were washed with PBS for removing extra aptamers prior to fluorescence imaging by an inverted fluorescence microscope (Olympus, IX53).

### Radiation

#### Irradiation procedure

4 MeV electron beam was provided to the samples by a Varian linear accelerator (LINAC) (Varian, Clinac 2300C/D, USA) following a dosimetric calibration. The cells were seeded in 12-well plates 48 h prior to irradiation. 24 h post seeding, the cells were exposed to the GNPs and/or AS1411/GNPs (0, 12.5 mg/L) for another 24 h. Before irradiation, the GNP containing medium was replaced with a fresh complete medium. The medium level was adjusted to 7 mm over the cells’ monolayer to place the cells at the approximate d_max_ of 4 MeV radiation beam. Irradiations were performed using a 25 × 25 cm applicator positioned at the top of dishes. The cells were set at 100 cm distance from the 4 MeV electron source. Irradiations were done in single fractions with a constant dose rate of 1 Gy per monitor unit. The cell culture plates were placed at the center of the electron beam to ensure that all the cells receive a uniform radiation dose. The radiation dose was also monitored and confirmed by using the parallel-plate ion chamber.

### Clonogenic survival assay

The effectiveness of radiation in presence of GNPs or AS1411/GNPs was assessed by measuring cell survival and renewal in clonogenic assay. Clonogenic assay is a gold standard assay for the measuring the destructive effect of radiation on cell genome. Following exposure to 0–6 Gy electron beams, cells were washed 3 times by PBS, trypsinized for 2–3 min, and re-suspended in complete medium, counted, and re-plated in six-well culture plates. The cultures were maintained incubated for 14 days without medium change. Cellular colonies were fixed using 10% formalin and then stained with 0.1% crystal violet for colony count. Surviving fractions (SF) were calculated relative to the number of starting plated cells and normalized to the non-irradiated control cells.

To evaluate radiosensitivity of mammospheres a previous protocol reported by Debeb et al. [31] was used with some modifications. Briefly, the mammosphere of MCF7 cells (7 days post culture) were trypsinized into single cells, counted and seeded in 96-well ultralow attachment plates with a density of 1000 cells/well for 24 h. After 24 h cells were treated with GNPs or AS1411/GNPs for additional 24 h. Then, the plates were exposed to different doses (0, 1, 2, 4 Gy) of 4 MeV beam. After radiation, the cells were incubated for 5 days prior to measurements. Spheres with a minimal size of 50 µm were counted using an inverted optical microscope.

### Statistical analysis

All experiments were carried out in triplicate and repeated at least for two times. The results are expressed as mean ± SEM. Statistically significant differences were tested by using one-way analysis of variance for the MTT and MTS assays and two-way analysis of variance for the clonogenic assays, both followed by Tukey’s post-hoc. Clonogenic assays in mammosphere culture were analyzed using Mann-Withney non-parametric test. P values less than 0.05 were considered as statistically significant.

## Result

### Characteristics of GNPs and AS1411/GNPs

**Fig. 1:**
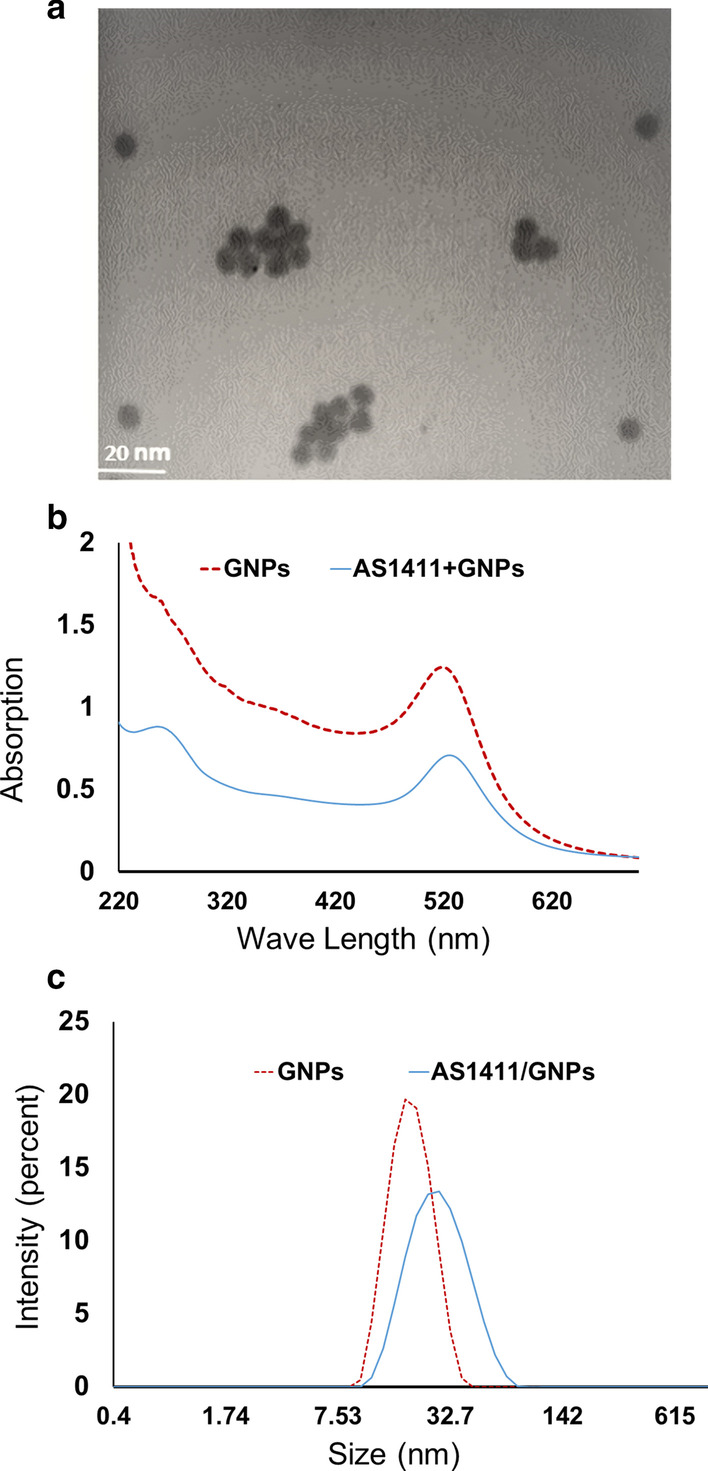
Characterization of synthesized GNPs and AS1411/GNPs. **a** The TEM image of the GNPs. The Mean of GNP sizes was 10 ± 0.7 nm. **b** UV–visible spectra of the GNPs and AS1411/GNPs. In the GNPs graph maximum absorption was observed at 518 nm and shifted in AS1411/GNPs spectrum to 525 nm. **c** Dynamic light scattering (DLS) image and the corresponding intensity distribution histograms of the GNPs and AS1411/GNPs

A transmission electron microscopy (TEM) image of the GNPs solution sample is presented in Fig. 1a. The average of GNPs diameter was 10 ± 1.5 nm. The GNP colloids were effectively spherically shaped. The UV–Visible spectra of the GNPs and AS1411/GNPs are shown in Fig. 1b. The optical UV absorbance peak at 518 nm was consistent with that of GNPs with a size of around 10 nm and the absorbance peak at 260 related to AS1411 aptamer was observed in AS1411/GNPs spectrum. In the spectrum of GNPs functionalized with AS1411 aptamer, a modest shift from 518 to 525 nm was observed; possibly due to the AS1411 conjugation [32]. Figure 1c shows the dynamic light scattering (DLS) images and corresponding intensity distribution histograms of the GNPs and AS1411/GNPs. The distribution and peak size of the AS1411/GNPs were changed compared to that of the GNPs alone. The DLS measurements showed an average size of 18.6 ± 5.4 and 25 ± 11.22 nm for the GNPs and AS1411/ GNPs respectively. The efficiency of coupling was calculated by measuring the changes in concentration of free AS1411aptamer before and after the conjugation. The average of molar ratio of aptamer to GNPs was equal to 72.16 ± 4.7.

### Effect of GNPs and AS1411/GNPs on the viability of MCF-7 and MDA-MB-231 cells

**Fig. 2:**
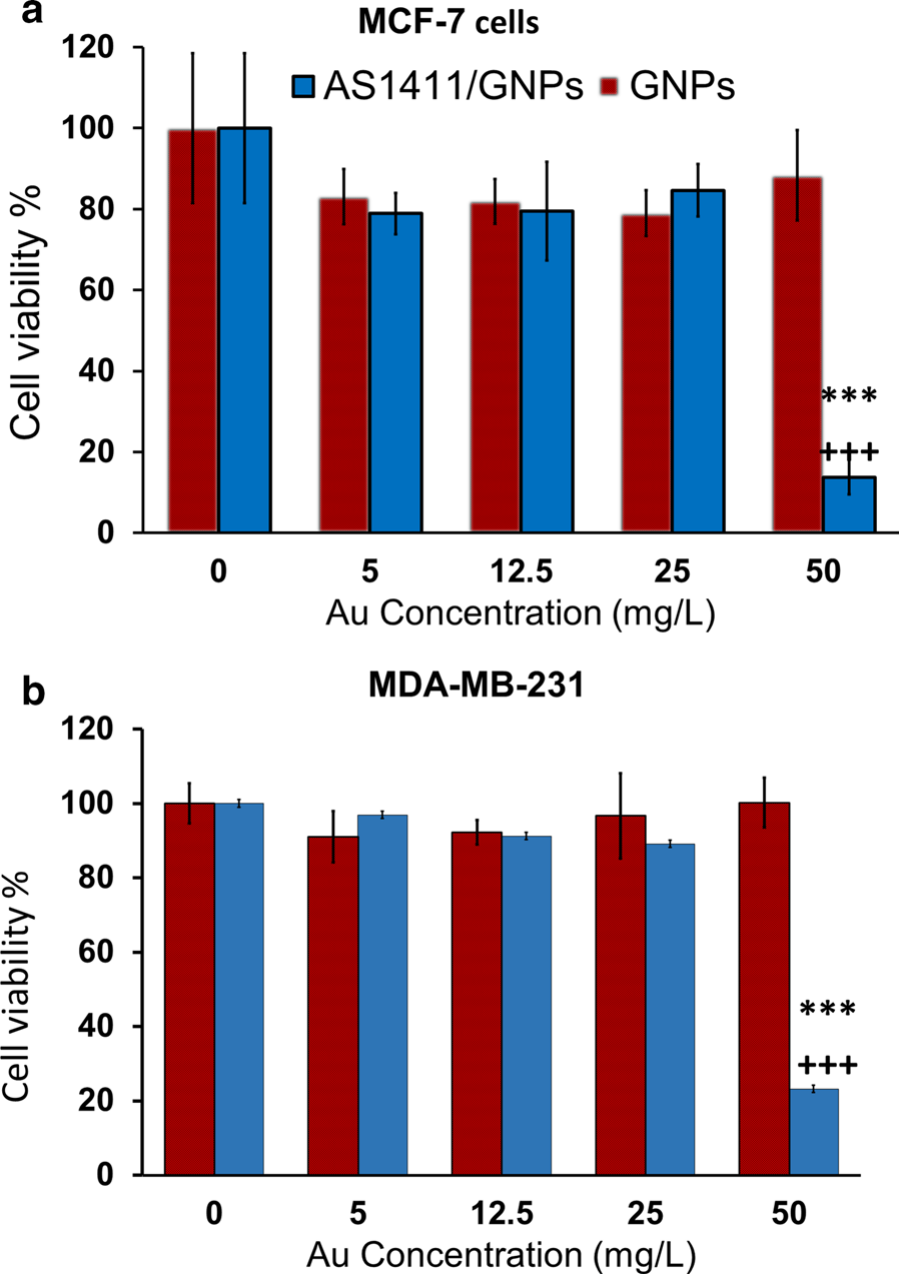
Cytotoxicity of GNPs and AS1411/ GNPs in MDA-MB-231 and MCF-7 cells. Cell viability of the MCF-7 (**a**) and MDA-MB-231 (**b**) treated with the GNPs and AS1411/GNPs was assessed by the MTT assay at different concentrations (5, 12.5, 25 and 50 mg/L) after 24 h treatment. ***p < 0.001copmared to control group, and +++p < 0.001compared to the GNPs group at the same concentration

MTT assay was performed to evaluate the cytotoxicity of the GNPs and AS1411/GNPs on the breast cancer cells. The effect of exposure to different concentrations (0, 5, 12.5, 25 or 50 mg/L) of the GNPs or equal amounts of GNPs in AS1411/GNPs preparation on the viability of MDA-MB-231 and MCF-7 breast cancer cells was studied (Fig. 2). The cytotoxicity was determined as the percentage of the viability of the treated cells normalized to the control group (GNPs concentration = 0). As Fig. 2 shows, MTT assay indicated no significant difference between GNP-treated and non-treated cells, despite the applied concentration (5, 12.5, 25 or 50 mg/L) for both of the breast cancer cell lines. When the cytotoxic effect of AS1411/GNPs was assessed at different concentrations of GNP content (5, 12.5, 25 and 50 mg/L), the results indicated that the viability of both cancer cell lines (MCF-7 and MDA-MB-231) was significantly (p < 0.001, p < 0.001) decreased at 50 mg/L concentration (Fig. 2a–b).

**Fig. 3:**
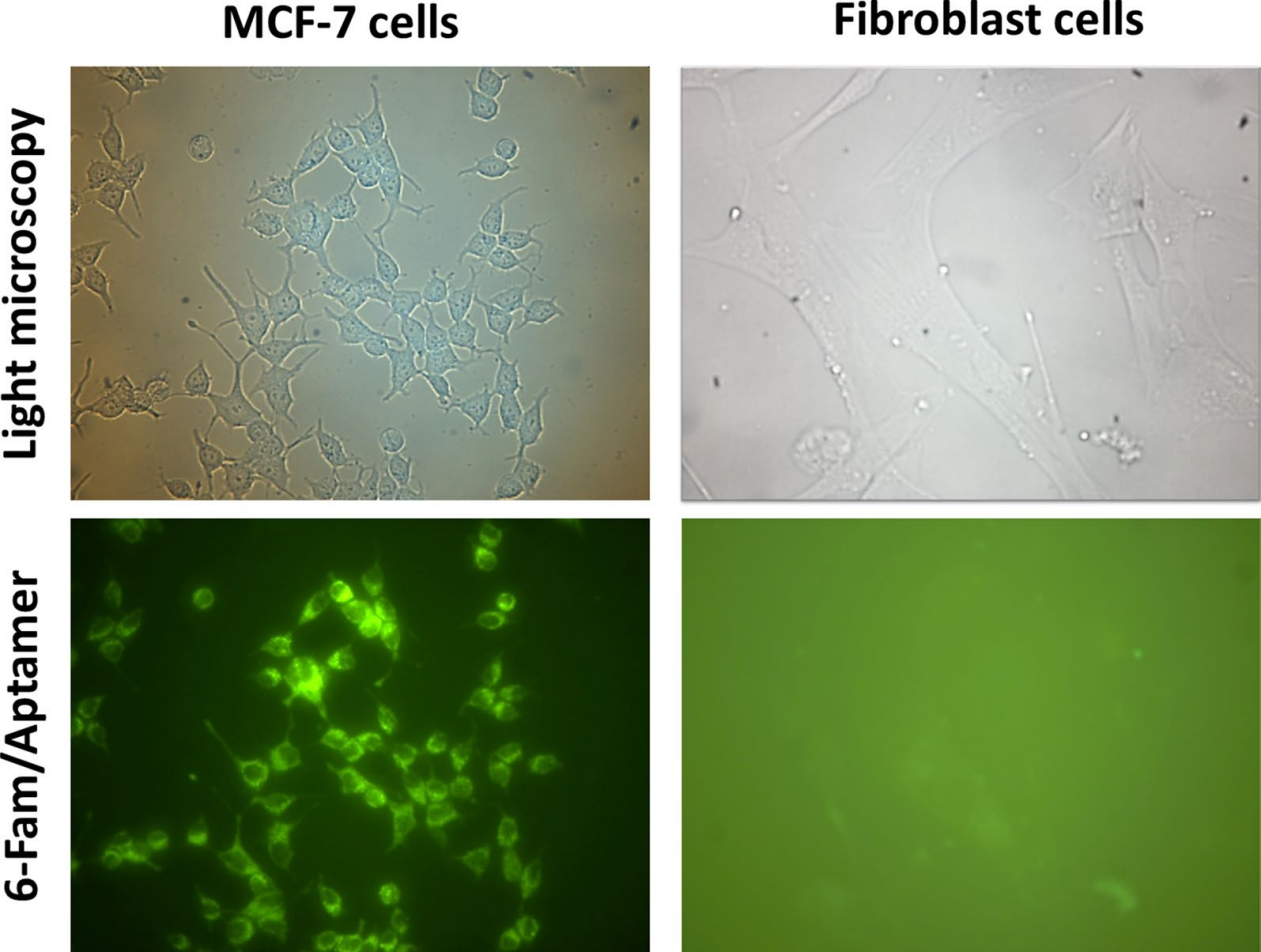
Fluorescent microscopy imaging of the cells treated with the AS1411 aptamer. Cellular uptake of 6FAM-labeled AS1411 aptamer in the MCF-7 (right panel) and fibroblast cells (left panel). Top row shows light microscopy and the bottom row shows the emitted fluorescent light (6FAM)

To confirm cancer cell specific uptake of AS1411 aptamer, the fluorescent microscopy imaging of MCF-7 and fibroblast cells treated with 6FAM-labeled aptamer (1 mM) was performed in 24 h after the treatment. The cellular uptake of 6FAM-labeled AS1411 aptamer in fibroblast, as the normal cells, was not observed, while a significant uptake was seen in MCF-7 cancer cells (Fig. 3).

### Atomic absorption spectroscopy studies

**Fig. 4:**
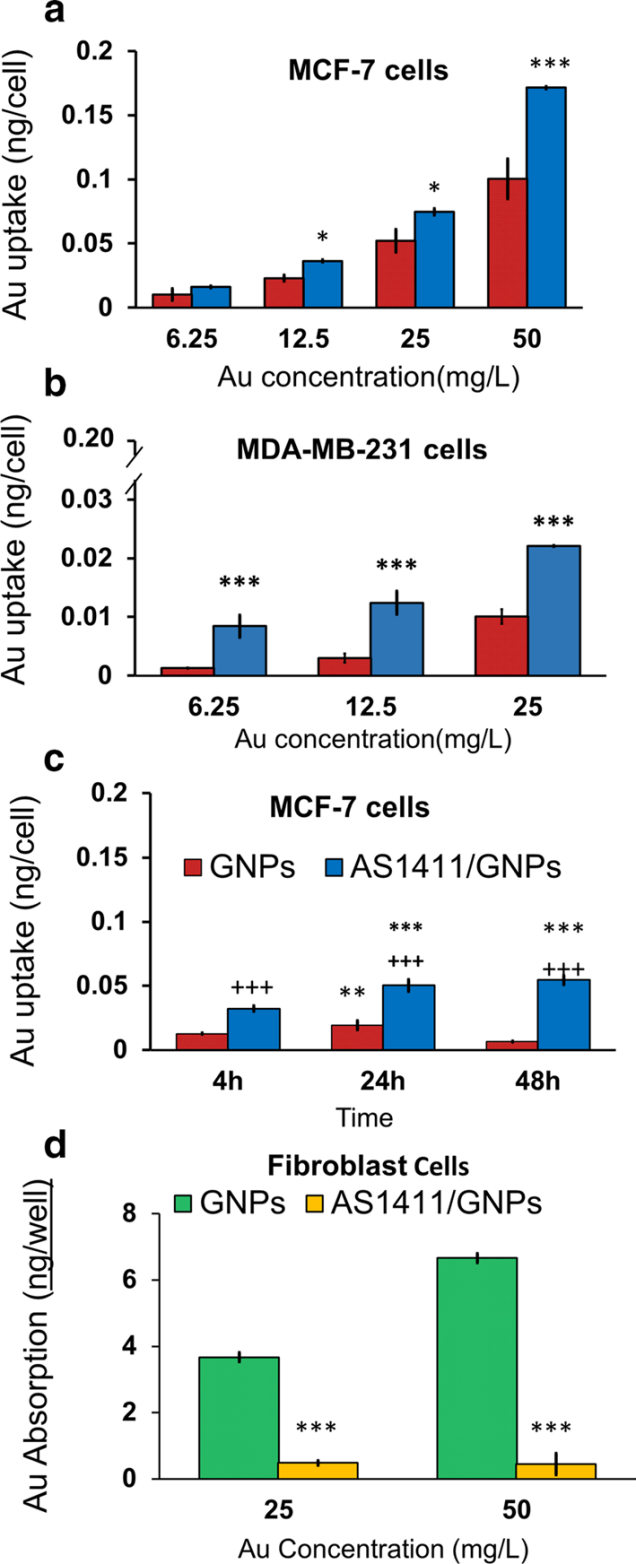
Au Cell Uptake following treatment of cells with GNPs and AS1411/GNPs measured by AAS. Au uptake at different concentrations (6.25, 12, 25 and 50 mg/L) of the GNPs and AS1411/GNPs studied in MCF-7 (**a**) and MDA-MB-231 (**b**) cells following 24 h treatment are presented. ***p < 0.001 and *p < 0.05 compared to the GNPs group at the same concentration. **c** Longitudinal study of Au uptake following treatment with GNPs and AS1411/GNP (both, 25 mg/L) in MCF-7 cells. Absorption of Au was measured after 4, 24 and 48 h of treatment. **p < 0.01 and ***p < 0.001 compared to same treatment in 4 h group, +++ p < 0.001 compared to GNP group at the same time. **d** Effect of aptamer A1411 conjugation on GNP uptake by fibroblasts. Fibroblasts as normal cells were treated with 25 or 50 mg/L of the GNPs or AS1411/GNPs for 24 h. ***p < 0.001 compared to GNP group at the same time

To assess the amount of Au uptake in the cells treated with the GNPs or AS1411/GNPs in 24 h after the treatment, the AAS study was used. Our results showed that compared with the groups treated with various concentrations of the GNPs, AS1411/GNPs groups showed higher Au uptake when the same GNPs concentration were applied. This increase was statistically significant for MCF-7 cells at 12.5, 25 and 50 mg/L concentrations (p < 0.05, p < 0.05 and p < 0.01, respectively) (Fig. 4a). For MDA-MB-231 cells, the increased uptake of Au was statistically significant at 5, 12.5, 25 mg/L concentrations (all, p < 0.001). When 50 mg/L concentration of the AS1411/GNPs was applied a few cells were remained due to extensive cell death in both breast types of cancer cells (Fig. 4b).

To obtain the optimum time of treatment, we also studied the Au uptake in MCF-7 cells exposed to 25 mg/L concentration of the AS1411/GNPs and GNPs in 4, 24 and 48 h after the treatment (Fig. 4c). This longitudinal study showed that in MCF-7 cells treated with GNPs, Au uptake was significantly increased after 24 h, compared to 4 h post treatment (p < 0.01) but did not change during the next day (48 h post treatment). In the GNPs group, the best effect was seen after 24 h. Cellular-targeting of Au using AS1411/GNPs preparation led to significantly higher levels of Au accumulation compared to the GNPs treated cells at 4, 24, and 48 h post incubation. 24 h incubation with AS1411/GNPs led to significantly increased Au cellular uptake compared to shorter incubation times, 4 h. There was no difference between the cellular level of gold when cells were treated with AS1411/GNPs for 24 h or 48 h.

We also measured the Au uptake in the fibroblasts as normal control cells following the treatment with the AS1411/GNPs or GNPs at 25 and 50 mg/L concentrations (Fig. 4c). At these concentrations, the cellular Au uptake was decreased in AS1411/GNPs treated cells compared with cells treated with the same concentrations of the GNPs (both, p < 0.001). For AS1411/GNPs groups, the Au uptake was not significantly increased by increasing the GNP concentration from 25 to 50 mg/L. We did not observed cell toxicity signs in fibroblasts treated with GNPs or AS1411/GNPs in different Au concentrations (25 and 50 mg/L) during the experiment time scale.

### Enhancing the effect of radiation with the GNPs and AS1411/GNPs

**Fig. 5:**
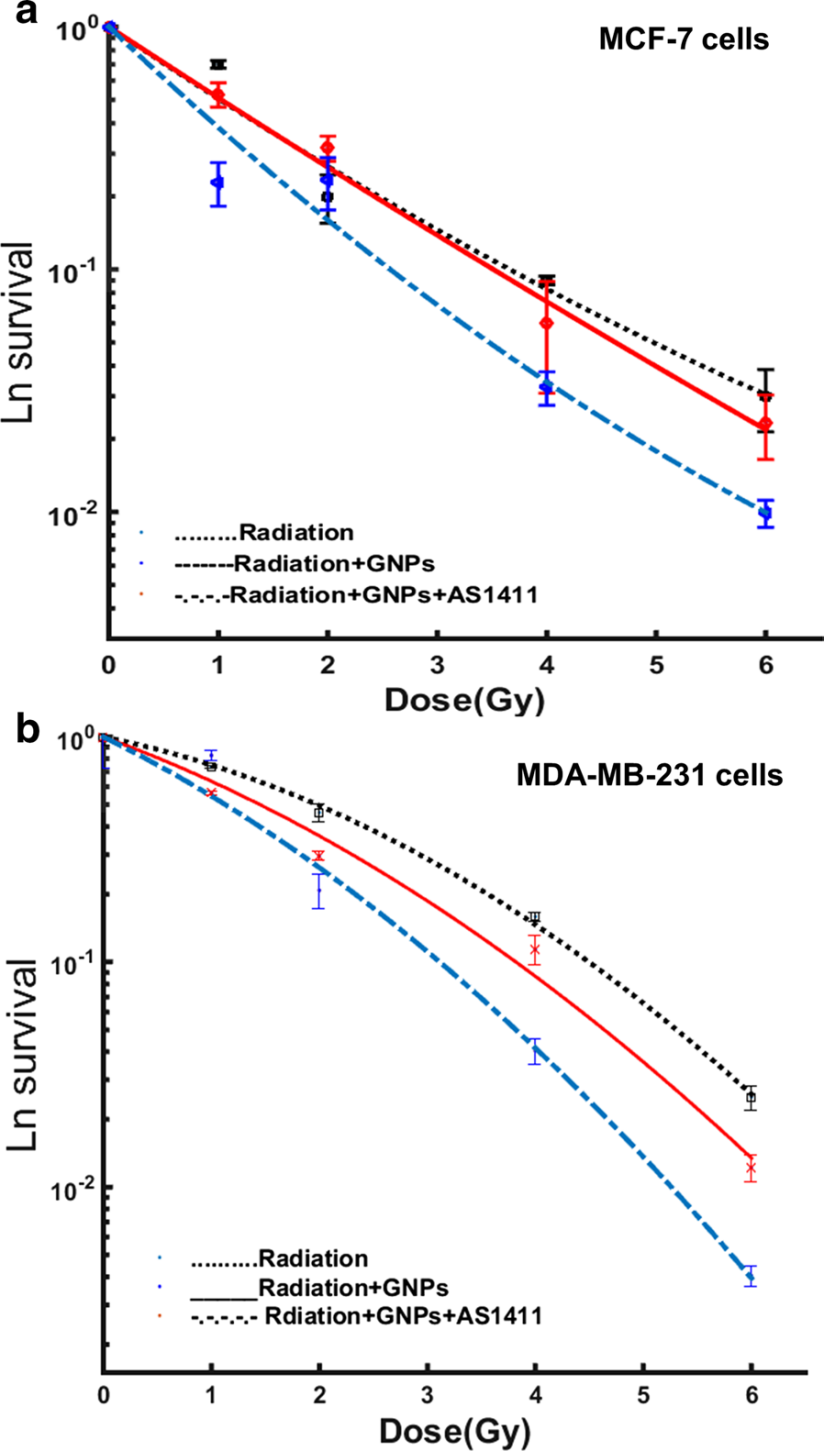
Radiation dose–response curves. Radiosensitization effect of the GNPs and AS1411/GNPs (12.5 mg/L) evaluated by clonogenic assay for the MCF-7 (**a**) and MDA-MB-231 (**b**) cells exposed to 4 MeV electron beams

The radiosensitizing effects of the GNPs and AS1411/GNPs were measured in different doses (0, 1, 2, 4 or 6 Gy) of 4 MeV electron beams. For this purpose, the MCF-7 and MDA-MB-231 cells were treated with 12.5 mg/L GNPs (or equal amount of GNPs in GNP/AS1411 preparation) for 24 h and then exposed to different doses of radiations. Figure 5 shows survival curve of MCF-7 and MDA-MB-231 cells in response to different radiation dose. In As1411/GNPs group the curve shifted to left side which showed a reduced survival or enhanced sensitivity to radiation by AS1411/GNPs. The dose enhancement by AS1411/GNPs was observed in both MCF-7 (Fig. 5a) and MDA-MB-231 (Fig. 5b) cells. The area under curve, as a representative of the mean inactivation dose (MID), was measured and the sensitizer enhancement ratio (SER) was calculated as the ratio of MID of non-exposed cells to those of gold -exposed cells [33]. SER was equal to 1.35 and 1.66 for MCF-7 and MDA-MB-231 cells. A linear quadratic curve fitted to data points was used to obtain both α and β components. An increase in both α and β components of the linear quadratic curve was observed (Table 1). Treatment with AS1411/GNPs, which showed a higher cellular uptake, led to significantly enhanced radiosensitization effect of GNPs in the cancer cells, while such radiosensitization effect was not achieved with the GNPs alone for the MCF-7 cells.

**Table 1:** The amount of sensitizer enhancement ratio (SER) and α and β components of the linear quadratic curve which was fitted to the data points of survival curve are presented

	Radiation	Radiation + GNPs	Radiation + AS1411/GNPs
MCF-7			
α (Gy^-1^)	0.688 ± 0.024	0.698 ± 0.025	0.995 ± 0.55
α (Gy^-2^)	− 0.0175 ± 0.015	− 0.0103 ± 0.006	− 0.0305 ± 0.011
R^2^	0.978 ± 0.017	0.955 ± 0.015	0.955 ± 0.026
SER	1	1.11	1.35*
MDA-MB-231			
α (Gy^-1^)	0.223 ± 0.039	0.401 ± 0.780	0.542 ± 0.370
α (Gy^-2^)	0.064 ± 0.058	0.053 ± 0.012	0.024 ± 0.050
R^2^	0.999 ± 0.078	0.993 ± 0.014	0.952 ± 0.059
SER	1	1.27	1.66*
Mammosphere			
α (Gy^-1^)	0.168 ± 0.062	0.092 ± 0.056	0.369 ± 0.178
α (Gy^-2^)	− 0.011 ± 0.009	0.009 ± 0.0039	− 0.033 ± 0.018
R^2^	0.999 ± 0.007	0.994 ± 0.007	0.994 ± 0.007
SER	1	1.04	1.91*

GNP s = gold nanoparticles; SER = sensitizer enhancement ratio

*p < 0.05 compared to radiation + GNPs

**Fig. 6:**
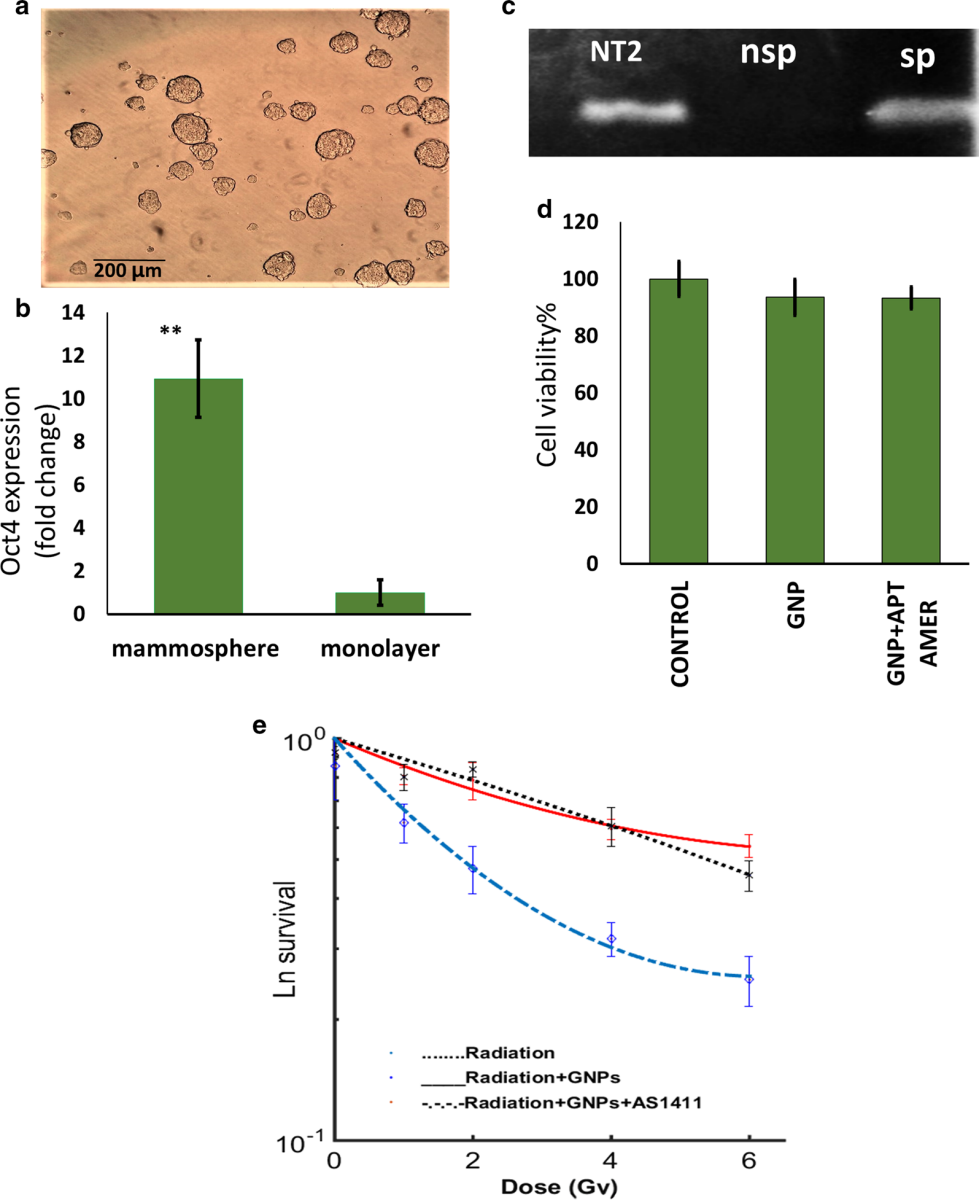
Radiosensitization in cancer stem-like cells enriched culture by AS1411/GNPs. **a** Picture of mammospheres obtained by the serum free culture of MCF-7 cells. **b** Real-time PCR data analysis demonstrating the increased expression of Oct4-a in mammospheres (***p < 0.001). **c** Gel electrophoreses of PCR products of OCT4-a expression in NT2 (positive control), monolayer (nSP) and mammosphere (SP) of MCF7 cells. **d** Viability of mammosphere cells treated with GNPs or AS1411/GNPs assessed by MTS assay following 24 h treatment with 12.5 mg/L Au. **e** Radiosensitization effect of the GNPs and AS1411/GNPs (12.5 mg/L) evaluated by clonogenic assay in cancer stem-like cells exposed to 4 MeV electron beams

The properties of cancer cells grown under mammosphere culture conditions (Fig. 6a) was studied by q-PCR. These cells showed higher expression of OCT4-a as a stemness markers of stem-like-cells when compared to the monolayer culture of MCF-7 cells (Fig. 6, B and C). This finding confirmed the enrichment of cancer stem-like cells in or 3D culture.

To explore the effects of the GNPs and AS1411/GNPs on the viability of the mammosphere-derived MCF-7 cells (without exposure to the radiation), we performed MTS assay (Fig. 6d). The MTS assay showed that 12.5 mg/L GNPs or AS1411/GNPs did not significantly reduce the cell viability in mammospheres.

The radiosensitivity of the MCF-7 cells grown under stem cell-enriching culture conditions was investigated by survival assays following exposure to increasing doses of 0, 1, 2, 4 and 6 Gy of 4 MeV radiation beams. The treatment with the AS1411/GNPs caused a significant radiosensitization effect, while such radiosensitization was not achieved with the GNPs alone in the mammosphere-derived MCF-7 cells. Similar to the monolayer cells, the area under the curve, was measured and SER was calculated. SER was equal to 1.04 and 1.91 for the GNPs and AS1411/GNPs, respectively. An increase in both α and β components of the linear quadratic curve was also observed (Table 1).

## Discussion

The major goal of this study was to see whether the conjugation of 10 nm GNPs with AS1411 aptamer would increase its uptake into the breast cancer cells and lead to enhanced radiosensitization when treated with 4 MeV electron beams. Our data indicated specific uptake of the aptamers into cancer cells and when conjugated with GNPs led to elevated levels of Au uptake by cancer cells. AS1411/GNPs radiosensitizer effect lead to more decline in survival fraction of irradiated cells when compared with the GNPs + radiation and the control groups; which may be attributed to increased Au uptake due to AS1411 aptamer-mediated cell entry (Figs. 3 and 5). Our findings were consistent with other studies which reported GNPs conjugation to folate led to greater GNPs uptake by Hela cells and enhanced radiosensitization when compared to the GNPs alone [34–36]. Moreover, several studies have showed that AS1411 aptamer increased the uptake of different nanoparticles into the cancer cells [12, 16, 37–39].

The cytotoxicity effects of the GNPs and AS1411/GNPs were evaluated. Among the different concentrations of GNPs and AS1411/GNP, our data showed that AS1411/GNPs 50 mg/L induced cell death while the lower concentrations or same concentration of GNPs did not reduce the cell viability which imply for enhanced Au uptake by aptamer (Fig. 2). Same cytotoxic potential of AS1411/GNP conjugate without any additional treatment in breast cancer cells has been shown. Near-infrared (NIR) light-absorbing hollow gold nanocages (AuNCs) functionalized with PEG and AS1411 (AS1411-PEG-AuNC) showed selective cellular uptake in breast cancer cells and the enhanced treatment efficiency of thermal therapy was demonstrated. They found a concentration depended effect of S1411-PEG-AuNC in MDA-MB 231 [40], although a bigger size and higher concentrations of nanoparticles were applied compared to our study.

Furthermore, the AS1411-linked gold nanostar particles had efficient uptake by cancer cells and effectively induced cell death [38, 41]. Previous studies showed that the biological activity of AS1411 is mediated by nucleolin which is highly expressed in cancer cells. Binding of AS1411 to nucleolin leads to efficient cellular internalization of nanoparticles and cell death induction [17, 42–44]. It is suggested that probably the structure of AS1411 contributes in this process, when the effective concentrations are achieved. Recently, biological effects of gold nanoparticles/AS1411 conjugates were studied by Kabiriani and colleagues. They suggest that the effect of AS1411 aptamer on cell proliferation may be mediated by neucleolin independent mechanisms. [39]. Beside the radiosensitization GNP/AS1411has been used to deliver other therapeutic agents to cancer cells. Kardani et al. used the complex of Au noanoprticle-AS1411 aptamer-antagomir 155 to decrease mir-155 in breast cancer cells [45]. Combination of AS1411, GNPs and photodynamic therapy have also shown therapeutic effects in the treatment of Hela cells [46]. In another study the combination of megavoltage radiation and AS1411 aptamer conjugated gold nanoclusters have been used as radiosensitizer and caused effective cancer cell death and a dose enhancement factor (DEF) of about 2.7 in clonogenic survival assay in breast cancer cells [47]. These data consist with our findings in different cultures including MCF-7, MDA-MB-231 and mammosphere derived cells. The reduced cell survival in AS1411/GNPs group may by the consequence of more effective Au uptake, or direct contribution of AS1411 into radiosensitization. Considering the various effect of AS1411 aptamer and its conjugates with GNPs, mechanisms others than increased GNPs uptake may be involved.

In recent years, many studies have been done on the radiosensitizing and dose enhancement effect of GNPs. GNP-induced radiosensitization is likely dependent on multiple variables including nanoparticles size and the shape [13, 48, 49] and its surface coating; radiation dose and the applied energy [7, 33, 50]. Most of the studies have emphasized on the radiosensitizing effect of GNPs and the dose enhancement of GNPs at kilovoltage (kV) energies of photon beams because of the dominance of photoelectric effect and its consequences at such energies [36, 51–53]. For electron beams at MeV energies, there are controversies between theoretical and experimental works. Considering the previous observations, regardless of the physical mechanisms of the effect of GNPs, studies have agreed on the radiosensitizing effect of GNPs in MeV energies [50, 52, 54] while dose enhancement for electron beams is negligible based on the physical characteristics. In vitro studies [33] have shown the specific radiosensitization in MDA-MB-231 cells, comparable with sensitizer enhancement ratio at kV and MeV energies. In vivo and in vitro studies by Chang et al. showed GNPs radiosensitizing effect at high energy electron beams [55]. To test our hypothesis that GNPs induce radiosensitization effect in the 4 MeV electron radiation beam condition, we made preliminary studies to measure the effect of 10 nm GNPs at different concentrations [56]. Our previous data showed that this sensitizing effect depends on GNP concentrations and the radiation dose. For electron beams, the achieved radiosensitization effect was higher than the predicted increase in physical dose, suggesting a strong biological component to be involved, interestingly, the Monte Carlo simulations have predicted the possible role of secondary electrons in radiosensitization of electron beams [57].

Gold may act as a biologically active agent that enhances the radiation damage by radiation beyond serving as an inert photon-absorbing element. The main mechanisms identified as being involved in the biological response of cells to GNPs radiosensitization are the DNA damage induction, production of ROS, cell cycle effects, oxidative stress, and potential interference with the bystander effects [58]. Since AS1411 aptamer and GNPs may enhance radiation effects through a number of distinct and overlapping mechanisms, the idea that the combination of the two agents additively or synergistically enhance radiation effects seems logical. Although in our experiment, this conjugation led to higher amount of Au uptake in cancer cells and consequently higher sensitizing effect was observed.

The study of cancer stem cells (CSC) has been made possible through an in vitro enrichment strategy called sphere culture [22]. We showed that the expression of OCT4-a as an essential gene for the self-renewal and pluripotency of stem cells was higher in mammospheres compared to the monolayers of MCF-7 cells. Similar results are reported from a study by Debeb et al. showing selectively expressed embryonic stem cell transcription factors of Oct4, Nanog, and Sox2 in 3D mammosphere cultures [31]. Radiation resistance of CSCs has been confirmed by several independent groups [18, 19, 31, 59, 60]. Phillips and coworkers reported that radiation resistance of breast CSCs was due to less reactive oxygen species production in response to radiation which imply for a high level of expression of free-radical scavengers [19]. Some efforts are made for targeting CSCs and overcoming their radiation resistance. Some investigations reported the efficiency of GNPs for targeting and overcoming the inherent therapeutic resistance of CSCs [61, 62]. Our study indicated that the efficiency of radiation in the mammosphere derived cancer stem cell-like cells was increased by AS1411/GNPs pretreatment when compared to GNPs alone. Regardless of whether the mammospheres can represent the CSCs population, they are a good 3-D model of the tumor cells.

## Conclusion

Gold nanoparticles delivery to the cancer cells was enhanced by AS1411 aptamer and led to enhanced radiation induced cancer cells death. The combination of our clonogenic assay and Au cell uptake results suggested that AS1411 aptamer has enhanced the radiation-induced cell death by increasing Au uptake, although other mechanisms may be also involved. This enhanced sensitization contributed to cancer stem cell-like cells to 4 MeV electron beams. This is particularly important for future preclinical testing to open a new insight for the treatment of cancers.

## Data Availability

All data obtained during the current study are available from the corresponding author on reasonable request.

## Abbreviations

GNPs: Gold nanoarticles
EPR: Enhanced permeability and retention
AS1411/GNPs: AS1411aptamer conjugated gold nanoparticles
CSC: Cancer stem-like cells
TEM: Transmission electron microscopy
DLS: Dynamic light scatterin
TCEP: Tris (2-carboxyethyle)phosphine hydrochloride
PTFE: Polytetraflouroethylene
PCR: Polymerase chain reaction
DMSO: Dimethyl sulfoxide
AAS: Atomic absorption spectroscopy
PBS: Phosphate-buffered saline
LINAC: Linear accelerator (LINAC)
SER: Sensitizer enhancement ratio
MTT: 3-(4,5-Dimethylthiazol-2-yl)-2,5-diphenyltetrazolium
MTS: 3-(4,5-Dimethylthiazol-2-yl)-5-(3-carboxymethoxyphenyl)-2-(4-sulfophenyl)-2H-tetrazolium
